# CoAR: Congestion-Aware Routing Protocol for Low Power and Lossy Networks for IoT Applications

**DOI:** 10.3390/s18113838

**Published:** 2018-11-09

**Authors:** Khadak Singh Bhandari, A. S. M. Sanwar Hosen, Gi Hwan Cho

**Affiliations:** Division of Computer Science and Engineering (CAIIT), Chonbuk National University, Jeonju 54896, Korea; khadak@jbnu.ac.kr (K.S.B.); sanwar@jbnu.ac.kr (A.S.M.S.H.)

**Keywords:** Internet of Things, LLNs, 6LoWPAN, RPL, objective function, routing metrics

## Abstract

The IPv6 routing protocol for low power and lossy networks (RPL) was designed to satisfy the requirements of a wide range of Internet of Things (IoT) applications, including industrial and environmental monitoring. In most scenarios, different from an ordinary environment, the industrial monitoring system under emergency scenarios needs to not only periodically collect the information from the sensing region, but also respond rapidly to some unusual situations. In the monitoring system, particularly when an event occurs in the sensing region, a surge of data generated by the sensors may lead to congestion at parent node as data packets converge towards the root. Congestion problem degrades the network performance that has an impact on quality of service. To resolve this problem, we propose a congestion-aware routing protocol (CoAR) which utilizes the selection of an alternative parent to alleviate the congestion in the network. The proposed mechanism uses a multi-criteria decision-making approach to select the best alternative parent node within the congestion by combining the multiple routing metrics. Moreover, the neighborhood index is used as the tie-breaking metric during the parent selection process when the routing score is equal. In order to determine the congestion, CoAR adopts the adaptive congestion detection mechanism based on the current queue occupancy and observation of present and past traffic trends. The proposed protocol has been tested and evaluated in different scenarios in comparison with ECRM and RPL. The simulation results show that CoAR is capable of dealing successfully with congestion in LLNs while preserving the required characteristics of the IoT applications.

## 1. Introduction

Internet of Things (IoT) is an emerging technology that has received great attention among researchers across the world. It has provided a promising opportunity to build powerful applications in areas such as industrial monitoring, healthcare, agriculture, smart grid, and transportation due to such features as being able to work with IP-based network and heterogeneous devices [1]. IoT enables thousands of intelligent devices such as sensors, and the convergence of information technology such as wireless communication, to be connected and integrated with computer networks [2]. Generally, sensing devices employed in the IoT network are typically resource constrained in terms of processing, battery power, memory, reliability, etc. In order to cope with those challenges, a number of solutions have been developed, for instance, emerging IPv6 over low-power wireless personal area networks (6LoWPANs) [3]. The IETF, IEEE, and ZigBee have developed various standards in recent years to enable resource-constrained devices to communicate effectively. Routing over low power and lossy networks (ROLL) group of IETF has provided protocol specifications for the routing protocol for low power and lossy networks (RPL) [4]. Using RPL, devices can communicate at the network level and be easily connected to the internet; therefore, it is considered the routing protocol for IoT.

RPL is designed to address resource constraints of embedded devices and to support the particular low power and lossy network (LLN) environments. It constructs the directed acyclic graphs (DAGs) according to routing metrics and constraints. Depending on the specific application, different routing metrics can be designed and adopted, which is also one of the issues in this paper. Moreover, in RPL a node selects the routing parent according to its objective function (OF) and establishes a routing topology as a destination oriented directed acyclic graph (DODAG). RPL separates the concept of objective function from core specification that enables the users to define routing strategies and local policies to fulfill the network and application requirements. So far, two OFs have been standardized in RPL that optimizes the parent selections towards the root node. However, both of them relying solely on a single metric as the routing decision metric and perform poorly in LLNs under heavy and dynamic traffic [5].

In most scenarios, different from an ordinary environment, the industrial monitoring system under emergency scenarios needs to not only periodically collect the information from sensing region, but also respond rapidly to some unusual situations. Although RPL has been mainly used for low rate traffic scenarios, it needs to be capable of handling the heavy traffic load. This is because during the events in a sensing region, nodes may generate and forward high traffic towards the root and it may cause congestion at parent nodes. Therefore, congestion-aware routing metrics and parent selection mechanism in RPL becomes critical.

In RPL specification [4], there is no explicit mechanism to detect and control the occurrence of congestion. Basically, RPL specifies a simple parent selection technique to avoid selecting parents with larger hop count or with a bad link quality. In fact, congestion in LLNs may occur at a particular node when the offered load exceeds the available capacity and, consequently, the received data has to be dropped. Besides this, congestion also happens due to the unfair utilization of the network resources. Generally, congestion occurs either at the sensor node or at the wireless link which is commonly known as node-level congestion and link-level congestion, respectively. Both types of congestion have a direct impact on quality of service (QoS) in IoT applications. Usually, node-level congestion is examined either by monitoring the buffer utilization or measuring the incoming and outgoing traffic rate, whereas link-level congestion is examined by monitoring the channel utilization. Therefore, in both cases, timely congestion detection and taking an appropriate step for avoiding its consequences is important, which is the main focus of this paper.

There are two general approaches to solve the congestion problem: resource control and traffic control [6]. In the resource control strategy, selection of an alternative non-congested or less-congested path towards the destination is employed. Unlike the resource control method, traffic control implies solving congestion by adjusting the traffic rate at source nodes or intermediate nodes. However, for an industrial monitoring system, reducing the sending rate of nodes is not acceptable in an emergency scenario IoT application. Therefore, considering these facts, the main objective of this paper is to design congestion-aware RPL (CoAR) that is suitable for dynamic and high traffic load. To achieve this, we mainly focus on three aspects that are the resource-control based congestion alleviation method, adaptive congestion detection mechanism and multi-criteria decision-making (MCDM) technique for parent selection method. The main contributions of this paper can be summarized as follows:

- This paper presents a congestion-aware routing protocol named CoAR, that addresses the congestion problem for IoT applications in LLN. It adopts the resource control strategy to fulfill the application requirements in emergency scenarios.
- This paper suggests a new objective function, known as a congestion-aware objective function (CoA-OF). CoAR implements it as the parent selection framework. It solves the parent selection problem within the congestion by implementing MCDM technique, which utilizes the multiple routing criteria.
- We propose a simple yet effective congestion detection mechanism based on an adaptive threshold. It allows the affected nodes to avoid transient congestion situation, and then to eliminate the persistent congestion situation, it triggers the parent change procedure to create the alternative path towards the root.
- We implement and analyze the proposed routing protocol, and conduct extensive simulations to validate the overall performance through Cooja simulator.

The rest of the paper is organized as follows. Section 2 presents a review of the related works. Section 3 explains the RPL and formulates the problem. Section 4 describes the proposed protocol in detail. Section 5 evaluates the performance of the protocol in the different scenarios and presents the results with a comparison. Section 6 discusses the key points of the work and some design limitations. Finally, Section 7 concludes the paper.

## 2. Related Works

In recent years, several works have evaluated the performance of the RPL standard specification and proposed some enhancements to potential shortcomings and open issues [7,8]. Although RPL greatly satisfied the requirements of LLN for IoT applications, some issues remain open for improvement such as the effect of congestion on QoS. Several new protocols have been proposed for wireless sensor networks (WSNs) [9,10] in order to avoid and control the presence of congestion. The proposed schemes differ in the way that they detect congestion, notify the occurrence of congestion, and the way they resolve the congestion problem. In many cases, a single metric cannot accurately perform the indication of congestion; therefore, a combination of specific metrics are taking into consideration. Some of the widely used metrics are channel load, buffer occupancy, packet transmission time, packet service time, packet loss and delay [11].

In such a scenario, when congestion is detected, the congestion notification must be broadcasted to allow for taking an appropriate action. This notification can be either implicit, the information can be incorporated in the data packets header that is piggybacked, or explicit, the information can be incorporated in separate control messages. Upon detection of congestion as well as according to application requirements, either the traffic or resource control method is applied [9]. In the case of traffic control method, the sending rate of a source node is reduced to a specific value in which the quantity of the injected packets into the network is suitably reduced that alleviates the congestion. Nevertheless, for some IoT applications, for instance, event-based and time-critical applications, the rate adjustment technique is not efficient and acceptable. On the other hand, in resource control, packets are forwarded to the destination node through alternative non-congested paths. This approach can be used when rate reduction methods cannot meet the application’s requirements, since reducing source traffic during a critical situation may violate application requirements.

The comparative study between traffic control and resource control protocols has been presented in [12]. The authors proposed an upstream congestion control protocol for WSNs, termed priority-based congestion control protocol in [13]. It periodically detects the congestion, using the ratio between packet service time and packet inter-arrival time as a congestion metric. This ratio is used to achieve exact rate adjustment with priority-based fairness. In [14], a hierarchical tree alternative path algorithm has been proposed to minimize congestion and ensure reliable data transmission in the event-based application through resource control. It tries ensuring application reliability during overload periods without reducing the sources rate while sending critical events. The authors in [15], formulated the problem of congestion control using game theoretic model at the media access control (MAC) layer of IEEE802.11p for wireless transmission in the network. In [16], the authors proposed fairness-based congestion control algorithm for WSNs to monitor the rate at which the node is receiving and sending data packets. Furthermore, data forwarding decision is based on the characteristic ratio and the queue size of a downstream node. However, most of the above-mentioned literature does not consider the specific characteristic of RPL and IEEE 802.15.4 standards. Recently, some research works conducted in this domain have suggested new congestion avoidance and control mechanism for the RPL network. A short review of the suggested mechanisms is given below.

In [17], the authors proposed a congestion control mechanism in an IoT environment. The proposed solution is based on the concept of bird flocking to guide the flow of packets in order to avoid congestion. The proposed work performs congestion control in a hop-by-hop manner in the network. The main idea is based on the attraction and repulsion zone of the focal node. Attraction zone contains the parents and child of next hop nodes of the focal node in two-hop bases, whereas the repulsion zone contains, the focal node, its parents, and child in one hop basis. It improves the packet reception rate. UDP message is used in order to estimate the buffer in the zones. However, in LLN characteristic, it may not be so obvious a choice, especially for time critical and event-based IoT applications because the focal node always eavesdrops (passive listening) the UDP messages, which may result in the inaccurate buffer calculation. Moreover, the focal node always eavesdrops to the channel, as a result, the radio is always on and therefore depletes the energy.

A game theory-based congestion control mechanism has been proposed in [18]. To detect the congestion on parent nodes, it uses the net packet flow rate as a metric which is the difference between the packet generation rate and packet service rate. In the protocol, as soon as the congestion is detected at a node, it broadcasts the congestion notification to its neighboring nodes. On receiving the message, a neighboring node triggers the parent change procedure. To carry out this procedure, the potential game theory method is used. If the congestion cannot be addressed successfully by the parent selection procedure, the parent node chooses a rate adjustment scheme. However, the rate adjustment scheme may drastically decrease the QoS of the network. Therefore, in IoT applications such as media streaming and emergency scenarios, this scheme may bring some negative impacts on desired requirements.

The authors proposed a duty cycle-aware congestion control for 6LoWPAN (DCCC6) protocol in [19]. The protocol controls the congestion using the presence of a radio duty cycle and regulates its operations accordingly. It detects the congestion based on a dynamic buffer. To reduce the congestion, it uses the modified additive increase multiplicative decrease (AIMD) mechanism. When a node detects the congestion, it informs the child nodes contributing to congestion to reduce the transmission rate based on the adjustment scheme. However, such a rate adaptation scheme may not be a desirable solution for our proposed IoT application scenario.

A queue utilization based RPL (QU-RPL) has been introduced in [5], which is based on the queue utilization (QU) for load balancing under heavy traffic. It takes the QU of neighboring nodes into consideration during the parent selection process. The QU factor is defined and uses in parent selection process to achieve the traffic load balancing. In addition, QU-RPL performs the queue adjustment to lower the probability of a node being selected as a next hop towards the root when its parent node is severely congested. When a node experiences a certain number of consecutive buffer overflows, the node broadcasts a DODAG information object (DIO) message that contains the congestion information. Upon receiving the congestion information, the node changes its parent that has less buffer utilization and lower hop distance to root. The protocol has been tested in a real testbed network. However, in this protocol, the congestion threshold is static, which may be appropriate for balancing the load in high traffic, but may not be for dynamic traffic or bursty traffic, which is essential in the scope of effective IoT service provisioning.

In [20], the authors proposed a game theory-based congestion control framework (GTCCF) for 6LoWPAN networks. The adopted non-cooperative framework takes into account node priorities and application priorities. GTCCF resolves the congestion problem by adopting a node’s sending rate using the Nash Equilibrium concept. Like other traffic control algorithms, GTCCF adjusts the node’s sending rate, which is also not appropriate for our proposed scenario. Moreover, during the selection of alternative less congested paths, it increases the control message overhead. The authors proposed a congestion avoidance multipath routing protocol for time-critical applications named CA-RPL in [21]. Additionally, the authors introduced DELAY_ROOT, a new routing metric that minimizes the average delay towards the DAG root. CA-RPL alleviates the congestion by distributing a traffic to different paths. However, the protocol degrades the network reliability because it does not aware the packet overflow that occurs at the nodes’ buffer. It also does not have a strategy to detect the congestion when it occurs. 

A new objective function based on fuzzy logic (OF-FL) has been proposed in [22], that provides the QoS guarantees for RPL. The main purpose of OF-FL is to provide a better characterization of the best path. It combines several routing metrics and provides a more comprehensive description of the quality of neighbor nodes in order to select the best forwarding candidate. OF-FL uses link quality, hop count, residual energy and latency as the metrics in the process of path optimization. However, it is not clear which metric should be optimized for the specific purpose. In [23], the authors analyzed the impact of objective function on the network topology using the link quality objective function (LLQ-OF) and OF0. However, the LLQ-OF does not consider the congestion problem. The authors studied the congestion control schemes and explained how the buffer state is monitored by the different congestion detection mechanisms in [24]. The authors further stated that monitoring the buffer state can be achieved either through a buffer threshold or through a periodic buffer where a threshold can be dynamic or static.

Recently, an adaptive parent selection mechanism in RPL for advanced metering infrastructure network named energy and congestion-aware routing metric (ECRM) has been proposed in [25]. ECRM considers the residual energy and queue utilization of neighboring nodes as a metric for parent selection criteria. It minimizes the power consumption and improves the packet delivery ratio of the network. The scheme adopts the resource control-based method to alleviate the congestion. Moreover, it also uses different metrics and constraints to calculate the rank and selecting the best parent. However, in ECRM, the congestion detection scheme is based on the static threshold. Additionally, in dynamic or bursty traffic scenario, such as industrial monitoring IoT applications, it might be prone to congestion situation due to its consecutive buffer overflow-based parent change policy.

The routing protocols mentioned in the literature are aimed at enhancing network performance in congestion scenarios. These protocols employed different congestion control method to obtain the application requirements. The majority of the protocols are focused on monitoring buffer capacity for deciding the occurrence of congestion, which may not be reasonable for event-driven IoT application. Therefore, the routing protocol should also observe the traffic trend to satisfy the aforementioned application objective. In order to alleviate the congestion, all the protocols have focused on either alternative route selection or rate adjustment method. In the rate adjustment method, the node limits the source traffic volume. The most challenging part of this method is in determining the right amount of rate reduction. This challenge becomes more critical for the LLN based IoT applications. In addition, a single routing metric for route selection has a severe effect of overloading in the network, that resulting in resource depletion. Therefore, the routing protocol should be concerned with utilizing the multiple metrics in LLNs. Based on this understanding, in this paper, we are encouraged to implement the resource control-based congestion alleviation and adaptive threshold-based congestion detection mechanism for emergency scenario IoT application. Furthermore, to facilitate the analysis of static threshold-based congestion detection scheme with the resource control-based method under different traffic scenario, we compare our proposed protocol along with RPL as well as with the ECRM. Table 1 enlists the basic comparative characteristic of the aforementioned protocols and proposed work.

## 3. The RPL and Problem Description

RPL is a distance vector routing protocol for LLNs compliant with the 6LoWPAN protocol. It operates at the networking layer and supports several link layer technologies, including IEEE 802.15.4.

### 3.1. RPL Control Messages

**Figure 1:**
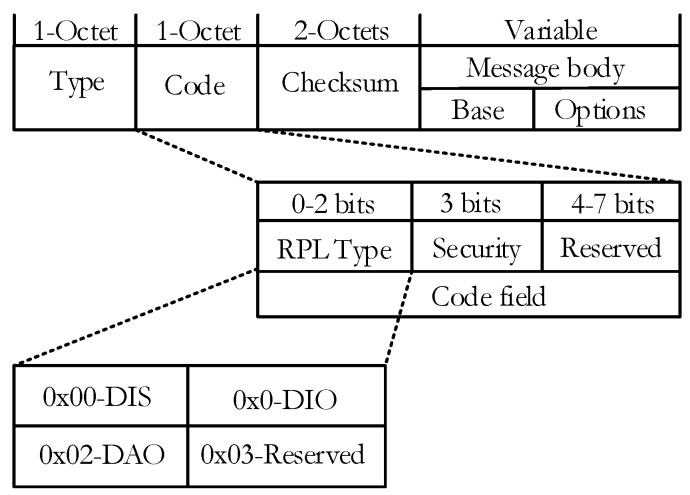
Control message format in routing protocol for low power and lossy networks (RPL).

RPL control messages are defined as a type of internet control management protocol version 6 (ICMPv6). The structure of the message is depicted in Figure 1, as in [7]. There are three types of control messages in RPL that are defined as follows:

- DIO (DODAG information object)
- DIS (DODAG information solicitation)
- DAO (destination advertisement object)

The DIO message is broadcasted by DODAG root to build a new DAG. It contains the network information that allows a node to discover an RPL Instance, learn its configuration parameters, and maintain the DODAG. The DIS message is used to solicit a DIO message from a node. This message is used to ask for DIO message from neighboring node and normally used when a node joins a stable network. The DAO message is sent by each node to propagate the route information towards the root. It is issued in a unicast manner and used to construct downwards routes (point-to-point and point-to-multipoint). Also, it contains the prefix reachability information.

### 3.2. DODAG Construction

In this phase, a root node initiates the DODAG construction by broadcasting a DIO message periodically. The neighboring nodes that reside within the communication range receive the message. Upon receiving the message, based on their OF, a neighboring node decides whether to join the DODAG or not. Each of the nodes that select the root node as a parent, re-broadcast the message. This process repeats at each node and continues until all the nodes in the network join DODAG to form a tree-structured topology. In RPL, the frequency of DIO message transmission is governed by the trickle algorithm in [26]. When a node decides to join the DODAG, it adds the address of the DIO sender to its candidate parent list and computes the rank. Along with this, the node ensures that its rank is greater than each of the parents which are listed. Once the node joins the DODAG, it forwards the message with updated information including the rank. A node receives the message, which is already associated with the DODAG, and it can either proceed with the message to maintain or improve the rank. Otherwise, it can discard the message. To avoid the routing loop, when a node changes its rank, it must discard all the listed parent nodes whose ranks are smaller than the newly computed node’s rank. If a node receives the message from multiple neighbors, it selects its parent from the candidate parent list based on the metrics or constraints defined by the objective function. In the case, when a node does not receive the message within a specific time or a new node wants to join the network, it first asks for the message from the surrounding nodes by sending a DIS message. Once a requesting node receives the message, it processes the message as explained before and constructs the route by sending a DAO message. For more detail refer to [4].

### 3.3. Communication Models

RPL supports three types of communication patterns that are point-to-point (P2P), point-to-multipoint (P2MP) and multipoint-to-point (MP2P). P2P is the routing between any two nodes in the DODAG. P2MP is the downward traffic transmitted from the root to the multiple nodes. On the other hand, MP2P is related to data collection traffic forwarded from child nodes towards the root node which is the main traffic flow in most of the IoT applications. The destinations of MP2P traffic is mainly DODAG root.

### 3.4. Route Repair

To assure the reliability of routing protocol, RPL supports two repair mechanisms, namely local repair and global repair. Local repair is to solve the issue locally instead of repairing the whole network. On the other hand, global repair can only be originated by the root node and fundamentally rebuilds the network topology by increasing the DODAG version number. When a node detects a network inconsistency such as a link/node failure or a local loop in a route, the node triggers the local repair. As a solution, it tries to find out the alternate path through the same ranked nodes although the recovery path may not be the optimal one.

### 3.5. Objective Function

RPL constructs the topology based on the OF. The OF defines how a node chooses an optimized path within an RPL instance based on routing metrics and constraints. It also defines the specific criteria of optimization, for instance, minimizing latency, hop count, etc. In route construction, the OF can combine different node and link characteristic routing metrics. Along with, it helps to determine the candidate parent set and preferred parent, so that the data traffic is transmitted through the preferred parent. Currently, there are two standard OFs used that are objective function zero (OF0) [27] and the minimum rank with hysteresis objective function (MRHOF) [28]. OF0 uses hop count as the routing metric, whereas MRHOF uses expected transmission count (ETX) as the routing metric for path calculation. The metric that OF uses is determined by the metrics in the DIO metric container. The RPL instance uses the OF to compute the rank for the node. The rank value is an abstract distance to the DODAG root within the DODAG version. In order to avoid the loop, the rank must monotonically increase from the root towards the child. The way of the rank is calculated depending on the OF.

### 3.6. Problem Description

**Figure 2:**
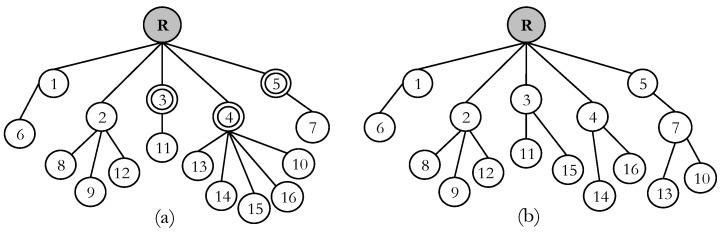
Destination oriented directed acyclic graph (DODAG) topology in RPL: (**a**) Parent selection problem; and (**b**) Topology construction based on the proposed scheme.

RPL was initially designed for light traffic network. However, IoT applications in an emergency scenario sometimes make prompt responses which result in heavy and dynamic traffic. In this situation, RPL cannot handle the traffic well. The consequence is the network faces severe congestion problem which results in packet loss and delay. As the resource constrained devices are considered in RPL, the majority of the packets are lost due to the buffer overflow in a congestion situation. The problem becomes more critical when the child node does not change their current parent and keep sending data traffic even though parent node is in serious congestion. Here, we classify the RPL congestion problem and consequences as follows: First, the routing metrics used in the default OFs do not reflect the congestion situation, therefore, nodes cannot be aware of congestion. In this circumstance, the affected node cannot indicate the presence of congestion. As a result, the child nodes do not change their current parent which is suffering from congestion. This is because the OFs allows each node to select their parent which has a reliable link (ETX) and shortest distance (hops) to root, regardless of congestion. In fact, the children of the congested node cannot know the congestion status of their subordinate parent. It is due to the fact that trickle is not aware of congestion and therefore cannot send congestion notification timely. In addition, RPL is implemented on resource constrained devices with a smaller queue size. Small queues start to overflow before traffic congestion becomes heavy enough to be recognized using ETX [5]. Second is the parent selection technique in the RPL among the node with the equal rank. To better understand the problem, consider the network topology as shown in Figure 2. Let us consider the rank of the nodes 3, 4 and 5 are equal and node 4 is overloaded compared to 3 and 5. These nodes are elements of the candidate parent set of nodes {10, 13, 14, 15, 16}. In this circumstance, the network’s resource is not properly balanced, which may create congestion in the future. Under low data traffic, node 4 may successfully forward data packets to the root node. However, if an event occurs in the sensing region, the node starts to send heavy traffic to their parents. In this situation, node 4 faces severe congestion because of the imbalance load. Thus, large number of packets are lost at buffer of node 4.

The packet loss occurs due to the default OF, because it selects the parent merely based on a single metric. Although ETX typically gives a reasonable estimation of the link status, using merely ETX to select the parent is not enough to balance the load in the network. When a node wants to join the network or change its parent, it selects the parent with the smaller rank. As a result, if node 4 propagates smaller rank, it attracts the nodes so that the nodes select it as their preferred parent without considering how much it is overloaded. Node 13 or 15 may change their parent after network topology reconstruction. However, this may not be an efficient solution; instead, the balance parent selection should be achieved at the beginning of the route construction. To address this problem, we design a new metric that selects the parent in a balanced way by checking the number of child nodes associated with the parent nodes.

## 4. Proposed Congestion-Aware RPL

This section describes the proposed CoAR. First, we formulate the routing metrics. Then, a novel congestion-aware objective function named CoA-OF is defined. For this, we utilize a technique for order preference by similarity to ideal solution (TOPSIS) of MCDM method which has been proposed by Hwang and Yoon in [29]. TOPSIS is one of the widely used methods that has been successfully applied to solving different problems in various applications [30]. The method combines multiple metrics to develop an objective function. It suggests that the best alternative should have the shortest distance from the positive ideal solutions and the farthest distance from the negative ideal solutions.

### 4.1. Routing Metrics

Several routing metrics are considered that reflect the probable congestion situation at node and link level. These metrics are used to find the best parent in term of congestion and define as follows.

Queue Utilization (QU): This metric refers to the neighboring nodes that have more free space in their buffers. It is defined as the buffer occupancy ratio of a node i to indicate the number of packets in the i’s queue out of the total queue size as in Equation (1). It is chosen for detecting and distributing information about congestion. It can serve as a simple and good indication of congestion and reflects the existing congestion situation at each node. Based on this matric, packets will be forwarded towards the idle or under-loaded parent. Here, we use the sliding window-based averaging to obtain queue occupancy.

(1) $$QU(i)=\frac{N_{p}Q(i)}{QS_{z}(i)}$$

where, *QU*(*i*) ∈ [0, 1], *N_p_Q*(*i*) is the number of packets in the queue of a node *i* and *QS_z_*(*i*) is the total queue size of *i*.

Expected Transmission Count (ETX): The *ETX* is known as a reliability metric that has been proposed in [31]. It is the expected number of transmissions required by a data packet to be delivered successfully as in Equation (2). This metric can help to select a data forwarding path that includes relatively high-quality communication link(s). It is an additive metric that adds the *ETX* of each link along the path to the destination. It is used to distinguish congested and/or lossy links. A link quality increases with decreases the value of the *ETX*.

(2) $$ETX(i,j)=\frac{1}{d_{ij}(data)\times d_{ji}(ack)}$$

where, *d_ij_*(*data*) represents the measured probability that a packet is received successfully by the neighbor and *d_ji_*(*ack*) is the measured probability that the acknowledgment is successfully received.

**Figure 3:**
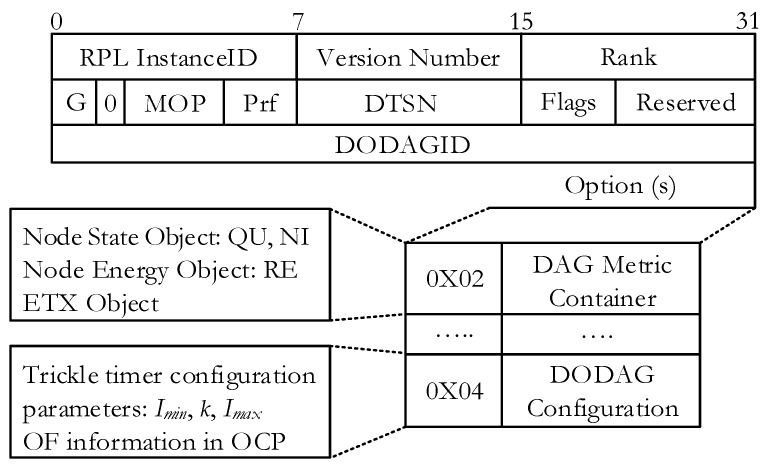
DODAG information object (DIO) message structure in CoAR.

Residual Energy (RE): This metric is used as the representative of network lifetime and permits to avoid selecting a node with low *RE*. The metric container uses the node energy object, as mentioned in Figure 3, to carry the *RE*. Likewise, node energy object contains the node type field which reveals the energy type of a node. In LLNs, how prone a node is to consume the energy depends on the node’s placement in the network as well as other factors. The *RE*(*i*) is defined as the difference between the maximum energy *MxE*(*i*) and the energy consumption *Ec*(*i*) by a node *i* as in Equation (4). Moreover, energy consumption can be estimated based on the power consumed by the node in each of the following possible states in which a node can operate. The states are the transmission (*T_x_*), reception (*R_x_*), *LPM* (low power mode, which basically means sleep mode) and *CPU* (radio is off but the system is active when processing information). In Contiki [32], an energy estimation module named *Energest* is used to obtain information about how long a system has been in different states. Additionally, power consumptions in these states can be easily obtained from the datasheet of the CC2420 chip. Energy consumption *Ec*(*i*) of a node *i* based on all the states with respect to time is obtained as in Equation (3).

(3) $$Ec(i)=T_{T_{x}}\times P_{T_{x}}+T_{R_{x}}\times P_{R_{x}}+T_{CPU}\times P_{CPU}+T_{LPM}\times P_{LPM}$$

where, *T_state_* and *P_state_* respectively, is the amount of time a node has spent in that particular state and the corresponding power consumed in that state at a particular unit of time.

(4) $$RE(i)=M_{x}E(i)-Ec(i)$$

Neighborhood Index (NI): *NI* is used to avoid the use of nodes that have many potential child nodes (data supplier) and few parent nodes (data recipients). Every individual node *i* in the network has its neighboring node. We define the neighboring node of *i* as *N_n_*(*i*) such that, each node *j* associated with *i* either as an upstream neighbor (*U_n_*) or as a downstream neighbor (*D_n_*). A *U_n_* is considered as a parent whereas a *D_n_* is considered as a child. It is worth noting that, this metric is not considered in rank computation rather it is used as a selection criterion of a preferred parent when the rank of candidate parents is equal. Even though the ranks of the nodes are equal, the number of children associated may be varied, and thus different criteria for being a parent. The reason for designing this metric is to balance the data traffic by taking into account the number of children for each candidate parent. Unbalance network problem has been addressed in the new draft for optimization of parent-node selection [33], where child node count metric is proposed. *NI* is the cost criteria which is used as a tie-breaking metric. We implement the storing mode of operation in this work in which the node stores the information of their neighbors, thus a node is able to calculate the number of child nodes. The *NI* is defined as the ratio of the number of *D_n_*(*i*) to the total number of all neighboring nodes *N_n_*(*i*) ∈ {*U_n_*(*i*), *D_n_*(*i*)} as in Equation (5).

(5) $$NI(i)=\frac{Number\;of\;D_{n}(i)}{Total\;number\;of\;N_{n}(i)}$$

### 4.2. Congestion-Aware Objective Function (CoA-OF)

As explained earlier, a single routing metric is not enough to achieve the application requirements; hence, the CoA-OF combines multiple routing metrics to address the congestion problem caused by unbalanced parent selection. CoA-OF uses TOPSIS which selects the best alternative based on the shortest distance from the positive ideal solution and the furthest distance from the negative ideal solution. Positive ideal solution has two criteria that are maximum benefit (for positive criteria) and minimum cost (for negative criteria), whereas negative ideal solution has a maximum cost (for positive criteria) and minimum benefit (for negative criteria). Shortly, based on the available criteria, the positive ideal solution comprises of all the best values whereas the negative ideal solution contains all the worst values.

Let us consider a node has a set of *m* candidate parent *P* = {*p_i_*, *i* = 1, 2, 3, …, *m*} with a set of *n* multiple routing metrics *R* = {*r_j_*, *j* = 1, 2, 3, …, *n*} for each parent. The weight vector *W* = {*w_j_*, *j* = 1, 2, 3, …, *n*} is composed of the individual weights (importance) for each routing metrics. Then, the congestion-aware parent selection problem can be represented by an evaluation (decision) matrix consisting of *m* alternatives (parents) and *n* criteria (routing metrics). To determine the most appropriate parent, three metrics (*ETX*, *QU* and *RE*) are considered as the parent selection criteria. In general, the criteria are classified into two types that are benefit and cost. The benefit criterion means that the higher value is better, whereas the cost criterion is the opposite. The TOPSIS process is carried out as the first steps by constructing an evaluation matrix *M* = *m* × *n* as follows:

(6) $$M=\begin{array}{c} \begin{array}{l} p_{1}\\ p_{2}\\ \vdots \\ p_{m} \end{array} \end{array}\;\begin{array}{c} \begin{array}{cccc} r_{1} & r_{2} & \cdots & r_{n} \end{array}\\ \left(\begin{array}{cccc} z_{11} & z_{12} & \cdots & z_{1n}\\ z_{21} & z_{22} & \cdots & z_{2n}\\ \vdots & \vdots & \cdots & \vdots \\ z_{m1} & z_{m2} & \cdots & z_{mn} \end{array}\right) \end{array}$$

where, *p_m_* are possible *m* parents (alternatives) among which decision makers (node) have to choose and *r_n_* represents the desired *n* metrics (criteria) with which alternative’s performance is measured. *z_ij_* is the value of *i^th^* alternative with respect to *j^th^* criteria, for all *i* = 1, 2, 3, …, *m* and *j* = 1, 2, 3, …, *n*. For our proposal, we have (*n* = 1, 2, 3) i.e., *r*_1_ = *QU*, *r*_2_ = *ETX* and *r*_3_ = *RE*.

The following remaining steps should be considered for generating the routing score among the candidate parents.

**Step** **1.** Normalize the decision matrix using the transformation formula: the normalized value $x_{ij}$ is calculated as in Equation (7).
  (7) $$x_{ij}=\frac{z_{ij}}{\sqrt{\sum_{j=1}^{n}{\left(z_{ij}\right)}^{2}}},\;j=1,2,3;\;i=1,2,\ldots ,m$$

Consequently, with this normalization, each criterion has the same unit scale. When the unit is varied for different routing metrics, for instance, some metric is measured in seconds, while other is measured in joule etc., the influence of some metrics may be neglected; therefore, it is necessary to normalize it, and hence, the normalized matrix *x_ij_* is obtained.

**Step** **2.** Calculate the weighted normalized decision matrix: the weighted normalized value is calculated as in Equation (8).
  (8) $$V_{ij}=w_{j}\times x_{ij},\;j=1,2,3;\;i=1,2,\ldots ,m$$
  where, *w_j_* denotes the weight of routing metrics *j* for all *j* = 1, 2, 3 satisfying $\sum_{j=1}^{3}w_{j}=1$.

It is well known that the weights of criteria in decision-making problem do not have the same mean and not all of them have the same significance. Therefore, in our proposal, we use the standard deviation method [34] to obtain the weights of the criteria. This method determines the weights of metrics in terms of their standard deviations as in Equation (9). These weights estimates are of relative importance for criteria.

(9) $$w_{j}=\frac{\sigma_{j}}{\sum_{n=1}^{3}\sigma_{n}}$$

where, *σ_j_* is the standard deviation of the values of *r_j_* determined as in Equation (10) and the mean is determined as in Equation (11).

(10) $$\sigma_{j}=\sqrt{\frac{1}{m}\sum_{i=1}^{m}{\left(x_{ij}-{\bar{x}}_{j}\right)}^{2}}$$

(11) $${\bar{x}}_{j}=\frac{1}{m}\sum_{i=1}^{m}x_{ij}$$

For all *j* = 1, 2, 3, the weights *w*_1_, *w*_2_ and *w*_3_ represent the importance of *QU*, *ETX* and *RE* respectively.

**Step** **3.** Determine the positive and negative ideal solutions: the positive ideal value set *A^+^* determines the best alternative whereas negative ideal value set *A^−^* determines the opposite as in Equations (12) and (13), respectively. These vectors are computed by using the following two relations.
  (12) $$A^{+}=\{v_{1}^{+},v_{2}^{+},\ldots ,v_{n}^{+}\}=\left\{\left(\max_{\forall i}\;v_{ij},j\in J^{\prime }\right)\left(\min_{\forall i}\;v_{ij},j\in J^{\prime \prime }\right)\right\},\;j=1,2,3;\;i=1,2,\ldots ,m$$
  (13) $$A^{-}=\{v_{1}^{-},v_{2}^{-},\ldots ,v_{n}^{-}\}=\left\{\left(\min_{\forall i}\;v_{ij},j\in J^{\prime }\right)\left(\max_{\forall i}\;v_{ij},j\in J^{\prime \prime }\right)\right\},\;j=1,2,3;\;i=1,2,\ldots ,m\;$$
  where, *J*′ and *J*″ are associated with the benefit criteria and the cost criteria respectively.

The positive ideal solution maximizes the benefit criteria and minimizes the cost criteria whereas the negative ideal solution maximizes the cost criteria and minimizes the benefit criteria. In our proposal, routing metrics *QU* and *ETX* are cost criteria (the smaller the value, the better the option), whereas *RE* is the benefit criteria.

**Step** **4.** Now, calculate the separation measures: the separation measures of each alternative (parent) from the positive ideal solution is given as in Equation (14).
  (14) $$D_{i}^{+}=\sqrt{\sum_{j=1}^{n}{\left(v_{ij}-v_{j}^{+}\right)}^{2}},\;i=1,2,\;\ldots ,\;m$$

The separation of each alternative from the negative ideal solution is given as in Equation (15).

(15) $$D_{i}^{-}=\sqrt{\sum_{j=1}^{n}{\left(v_{ij}-v_{j}^{-}\right)}^{2}},\;i=1,2,\;\ldots ,\;m$$

**Step** **5.** Calculation of the relative closeness to the positive ideal solution: the relative closeness of the alternative *p_i_* can be expressed as in Equation (16).
  (16) $$C_{i}=\frac{D_{i}^{-}}{D_{i}^{+}+D_{i}^{-}},\;i=1,2,\;\ldots ,\;m$$
  where, *C_i_* is the value of interval [0, 1], the closer the value is to 1 the higher the priority of the *i^th^* alternative.

Here, we grade the best alternatives (parents) according to the *C_i_* value. The highest grade represents the best rank which is equivalent to the RPL objective function rank. Using TOPSIS to select the preferred parent in each node is considered as an appropriate method since the computation is straightforward and the complexity of this method is an *O*(*m* × *n*). Here, *m* represents the number of neighbors of each nodes and *n* represents the number of metrics (*n* = 3). In the same way, calculation complexity is linear and compatible with *m* and *n*. Hence, the calculations are simple and the costs are not high. As a result, there will be no problem with resource constrained devices. It is worth mentioning that the complexity of other protocols relies on the number of metrics comprised. Finally, we compute the routing score of alternative parents as in Equation (17).

(17) $$\eta (i)=C_{i}\times \phi$$

where, *η*(*i*) is the routing score and used to make the decision in parent selection, and *ϕ* is a positive integer (e.g., 10 or 100). For our experiments, we have used 10. This score can lead to a better decision in the network within congestion.

### 4.3. Parent Selection Scheme

When a node wants to change its parent due to some inconvenience, for instance, buffer overflow at the parent node, or wants to join the network first time, CoAR selects the most suitable one between the candidate parent set (*C_pars_*) based on the routing score. Moreover, it checks the node’s neighbor density through *NI* metric during the selection of a parent when the routing score is equal. Algorithm 1 summaries the proposed parent selection mechanism, which makes use of CoA-OF and *NI* metrics to choose the best parent. The algorithm first creates the set of candidate parents as a list. After that, it finds the routing score of all the elements in the list and sorts them in descending order. For each node in the candidate parent set, it checks the routing score as the initial criteria (line 4). If the score of the candidate parent that currently selected is maximum, then the candidate parent node is selected as the preferred parent. However, if the candidate parent in the list that has equal routing score, it checks the neighbor density (line 9) and the one with the smallest *NI* value is selected as the preferred parent. In this way after checking all the nodes in the list of candidate parent, the algorithm returns the preferred parent as the selected parent.

**Algorithm 1:** Preferred parent selection mechanism

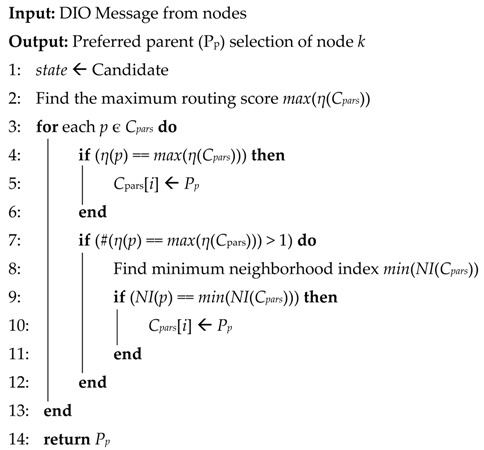



### 4.4. Congestion Detection

Measuring the congestion situation at each intermediate node, CoAR uses a congestion detection mechanism based on the current queue occupancy and observation of present and past traffic trend. In an IoT application scenario, sometimes congestion is happened because of the burst traffic. When a burst occurs around a node, a significant number of packets will enter the queue of a node at a given time period. A node is then congested in terms of occupied buffer space. CoAR handles this situation by using an adaptive buffer threshold. The dynamic trend with which node queues grow or shrink depends on the difference of the incoming and outgoing traffic rate through the specific node *i* at time *t*. Thus, buffer queues grow with the increase of incoming packet flow than the flow of outgoing packets. The incoming traffic rate $\left(\lambda_{in}\right)$ and outgoing traffic rate $\left(\lambda_{out}\right)$ of a node are not constant with time and its increment or decrement is based on the MAC parameters, backoff time of CSMA operation, and number of active nodes.

To determine a congestion situation adaptively, the term congestion degree (CD) indicates the changing tendency of the traffic load in a period of time and calculated based on the sliding window. The *CD* is defined as the ratio of incoming traffic rate over the outgoing traffic rate within a unit time interval in each node *i* as in Equation (18).

(18) $$CD(i)=\frac{\lambda_{in}(t)}{\lambda_{out}(t)}$$

We use an exponential weighted moving average (EWMA) filter to reduce measurement fluctuations at each node *i* to monitor the traffic rate $\lambda_{in}(t)$ and $\lambda_{out}(t)$ as in Equations (19) and (20).

(19) $$\lambda_{in}(t)=(1-\beta )\times \lambda_{in}(t-1)+\beta \times \lambda_{in}^{\prime }(t)\;$$

where, *β* is the smoothing factor such that 0 < *β <* 1 and $\lambda_{in}(t)$, $\lambda_{in}(t+1)$ and $\lambda_{in}^{\prime }(t)$ are expected average incoming traffic, old average incoming traffic, and recent incoming traffic rate respectively.

(20) $$\lambda_{out}(t)=(1-\alpha )\times \lambda_{out}(t-1)+\alpha \times \lambda_{out}^{\prime }(t)$$

where, *α* is the smoothing factor such that 0 < *α* < 1 and $\lambda_{out}(t)$, $\lambda_{out}(t-1)$ and $\lambda_{out}^{\prime }(t)$ are expected average outgoing traffic, old average outgoing traffic, and just transmitted traffic rate respectively, and *t* is a unit time. A large value of smoothing factor *α* and *β* reduces the level of smoothing and prioritize to weight to current measurement of traffic. whereas, a value of *α* and *β* close to zero gives greater smoothing effect and less responsive to current changes in traffic.

Moreover, in the process of determining the congestion degree, Equations (19) and (20) are updated periodically per incoming and outgoing packet basis. In our work, incipient congestion is detected by using a simple thresholding technique. Queue measurement begins when the queue occupancy of each node traverse 50% of the total queue size. Thus, this threshold is set as a warning line denoted as *γ*. At this instance, the node checks the congestion degree through which the adaptive congestion threshold (*A_τ_*) of buffer for the given traffic rate considering the remaining queue size can be achieved as in Equation (21).

(21) $$A_{\tau }=\gamma +\frac{1}{CD}\times (remaining\;queue\;size)$$

The *A_τ_* specifies the anticipated queue level at *t +* 1 based on the current traffic. This *A_τ_* value allows the affected nodes to tolerate burst traffic. Therefore, the threshold value is set to maximum when incoming traffic is zero or lower than outgoing traffic. Moreover, the threshold value is inversely proportional to the incoming traffic, hence it decreases as the incoming traffic increases. Let us consider that the incoming traffic is double than the outgoing traffic and total queue size is 48 packets. When the 50% of the queue (24 packets) is full the node begins the queue measurement. First, it checks the congestion degree, in this example the queue filling ratio is double that means when a single packet will exit from queue two packets will enter in the queue. Thus, *CD*(*i*) = ½ and the remaining queue size is 24 packets. The congestion threshold can be calculated according to Equation (21) i.e., *A_τ_* = ½ × 24 = 12 packets. Now, with this ratio node can hold 12 packets in the next time interval and total packets in the queue will be 36. Thus, in this example, the threshold (*A_τ_*) is set to 75%. When the queue occupancy exceeds the threshold, a node triggers the parent change procedure to alleviate the congestion problem. By using the adaptive threshold-based congestion detection mechanism the proposed algorithm is able to face the congestion situations successfully.

### 4.5. Congestion-Aware RPL Description

The proposed CoAR is designed to use the network resource effectively and utilizes the alternate parent selection strategy of congestion alleviation method. In order to achieve the design objective, the node periodically checks the congestion situation every time interval ‘*T_CC_*’. The node checks the congestion conditions by monitoring the buffer. When the node’s *QU* is higher than the *A_τ_* it enters the congestion state and the congested node broadcasts the DIO to its children. As CoAR implements the implicit congestion notification, therefore the congestion information is piggybacked in the DIO packet and thus reduce the control message overhead. Upon receiving the DIO message with congestion notification the child node undertakes the parent change process and CoA-OF resolves the parent selection problem within the congestion. It selects the alternate non-congested or less congested parent node and then forwards data packets through it. However, the DIO transmission scheme in RPL is regulated by the trickle algorithm where the frequency of sending DIO message is decided by the trickle timer.

The trickle timer is set to minimum interval length (units of time) *I_min_* and when the interval expires, trickle doubles the interval length until the time specified by *I_max_*. Therefore, it is worth noting that the RPL does not provide congestion information in a timely manner due to its long interval length since the timer is reset to a minimum only when inconsistency occurs in the network. To solve this problem in CoAR, the operation of the trickle algorithm is modified such that when the congestion occurs at the parent node, the trickle timer is reset to *I_min_*. This reset strategy allows nodes to promptly propagate its information during congestion so that child nodes can quickly select an alternate parent. Algorithm 2 shows the congestion alleviation procedures of CoAR.

**Algorithm 2:** Congestion alleviation operation of CoAR

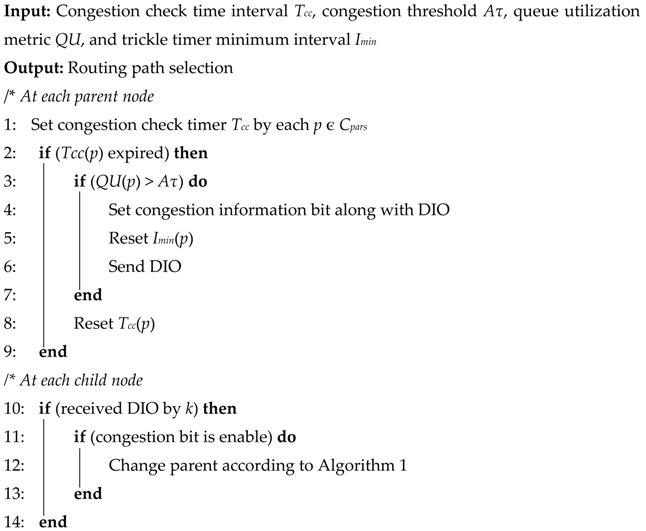



DIO message carries relevant network information such as configuration parameters and it can be extended with the use of its options’ field. The DAG metric container object that exists in the options’ field of the DIO packet format carries the multiple chosen routing metrics and constraints. The routing metric/constraint objects represent a metric or a constraint of a particular type. They may appear in the metric data field of DAG metric container in any order. A tie-breaking metric can be used according to their precedence defined by their *prec* filed values in object format [35]. For congestion notification, we use a *CN* bit in the DIO message. The DODAG configuration option is generally configured and allowed to be modified by the root. It is used to report the configuration information through the DODAG. The simple structure of the DIO is shown in Figure 3.

## 5. Performance Evaluation

In this section, we evaluate the performance of CoAR. The congestion alleviation mechanism employs a resource control method. This method demands different scenarios to study their behavior broadly. Therefore, we evaluate the proposed protocol through extensive simulation tests and implement the simulation environments as close to the real conditions as possible. At first, we analyze how the parameter settings in CoAR impact the network performance in different scenarios. Then, to verify that the proposed protocol is suitable for LLNs in emergency scenarios, we examine and compare the performance of CoAR with ECRM and RPL in several performance metrics.

### 5.1. Simulation Environment and Settings

**Figure 4:**
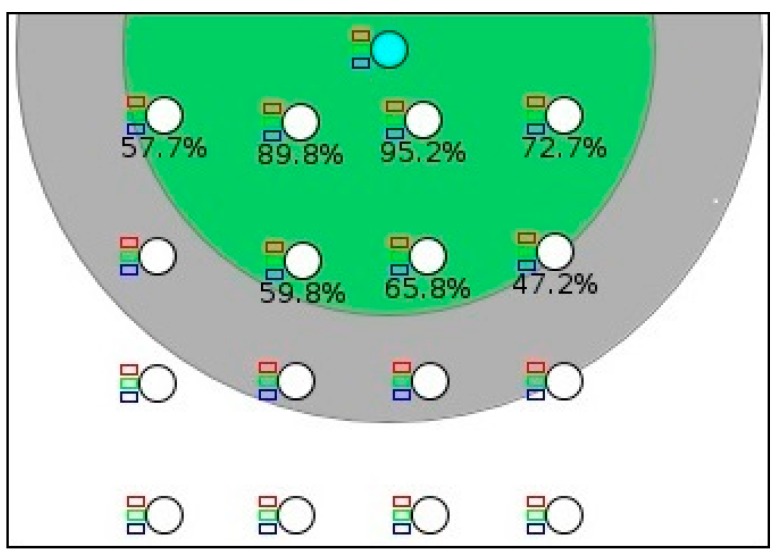
Example topology of the network.

The proposed protocol is designed for IoT applications considering emergency scenarios that comprise any critical situation such as chemical and gas leakage, electrical short circuit, fire outbreak and so on. For instance, a typical application of an emergency scenario is a monitoring and warning system in the industry, home or in a building. A number of sensor nodes are deployed in the monitoring region and build a network for the industrial monitoring system. The nodes continuously response about the situations to a root node either directly or hop-by-hop. For this, 16 Tmote sky devices were deployed randomly that are composed of a CC2420 radio transceiver with a data transfer rate of 250 kbit/s using IEEE 802.15.4 MAC and physical layer specification. The simulated monitoring network comprised of one server node acting as a DODAG root node. The root node was located at the top of the network field and the other nodes were spaced 50 m from each other as shown in Figure 4. The proposed protocol was implemented in the Contiki 2.7 [32]. Contiki is an open source operating system for the IoT. Simulations were conducted with Cooja [36]. Cooja is a flexible Java-based cross-layer simulator that is part of the Contiki OS. In the simulation, *T*_CC_ was set to 256 clock ticks. A constant bit rate (CBR) traffic model with a packet size of 56 bytes was used. Each simulation run lasts for 8 min. During the simulation, the source nodes start generating data packets after 60 s. The source nodes are selected independently and randomly for each simulation run and kept them steady during the period. For our experiments, we have set (*α, β*) *=* 0.4. Each simulation run has been performed 10 times and average results have been extracted and presented. The rest parameters used in the simulations are given in Table 2.

### 5.2. Comparative Analysis

**Figure 5:**
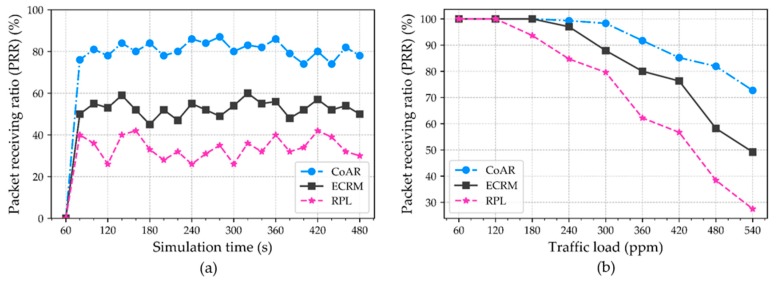
Packet receiving ratio (PRR) on the root: (**a**) Data transmission over time, PRR with respect to the different time intervals; and (**b**) PRR with respect to the varying traffic load.

**Packet receiving rate (PRR).** It is defined as the ratio between the number of packets received by the root and the number of packets sent by the source nodes. Due to the high data traffic from several sensing nodes, the packets may converge on a particular intermediate node and that results in the packet drop because of the buffer limit being exceeded. This situation leads to a fewer number of packets being received at the destination and, hence the PRR decreases. However, PRR is measured in the different time intervals based on the average over periods of 20 s. Figure 5a shows the PRR of CoAR, ECRM, and RPL with respect to the varying time. The result depicts the trend of the metric in the protocols is fluctuating over the time. Yet, CoAR obtains the smaller PRR fluctuation than ECRM and RPL when congestion occurs. In contrast, RPL lost most of the packets compared to CoAR and ECRM. This was expected because RPL does not aware of congestion in the network. The reason behind the higher PRR in CoAR is that it detects the congestion proactively and can bypass the affected nodes by selecting less or non-congested parent node.

We further analyze the PRR with respect to the varying traffic rate. For this, different traffic load is offered that is ranged from 60–540 packets per minute. The Figure 5b shows that the trend of the PRR at root node decreases with the traffic load increases. CoAR achieves a higher PRR compared to ECRM and RPL under high traffic load. Besides, CoAR maintains the maximum and minimum PRR of 100% and 72.7%, whereas, ECRM maintains 100% and 49.2% respectively. On the other hand, RPL decreases the PRR dramatically as the traffic load increases and reached a minimum of 27.5%. CoAR outperforms the ECRM and RPL by an average of 8.9% and 20.7%, respectively. Meanwhile, it is also more reliable against the gradual growth of the traffic load. Thus, it is shown that, at high traffic load, CoAR achieves higher PRR by exploiting the network resources in an effective manner.

**Figure 6:**
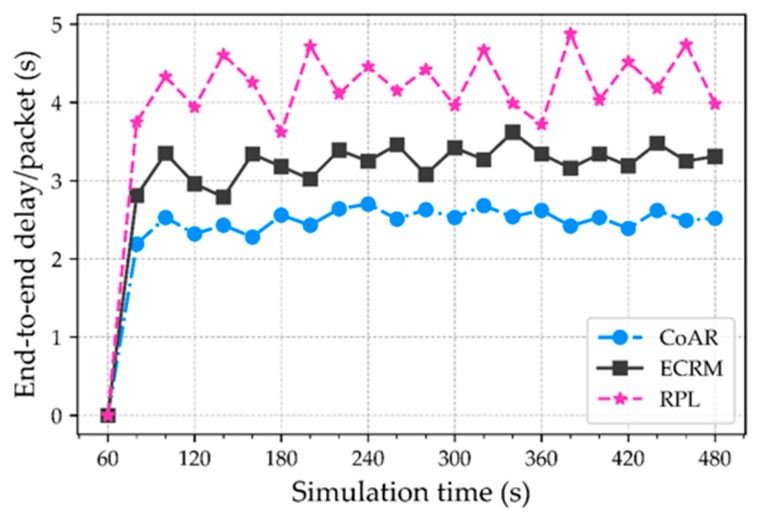
Average end-to-end delay per packet over time.

**End-to-end delay.** This metric is defined as the average time interval between packets generated at a sensing node until it successfully received at the root node. We use the warning line in queue monitoring to ensure the packets have a low delay. Figure 6 shows the evaluation of an average delay. The result shows, CoAR ensures the lowest packet delivery delay compared to the protocols. The delay of the RPL is significantly higher than CoAR and ECRM. This was expected because the RPL is unable to make a timely delivery of the data packets and needs to wait a long time in the buffer during congestion. In contrast to this, the CoAR can detect the occurrence of congestion and performs better. Because after detecting congestion, traffic is always routed through the non-congested or least congested parent in CoAR, so that packets are not queued in nodes buffering for a long time.

**Figure 7:**
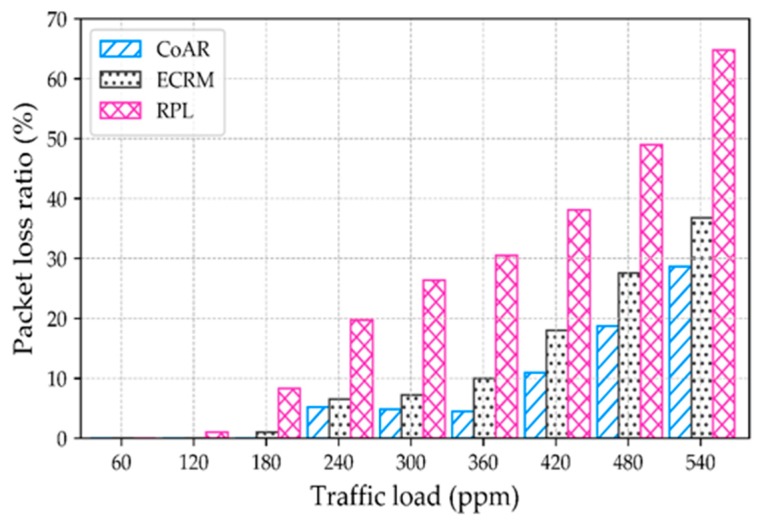
Packet loss with respect to the traffic load.

**Packet loss ratio.** Packet loss ratio is the ratio of the total number of data packets lost due to the buffer overflow to the total number of packets sent. Figure 7 compares the packet loss ratio with respect to the increasing traffic load of the protocols. The result shows the packet loss ratio increases with increases the traffic load. However, CoAR loses fewer packets at the buffer than others. When the data rate is high (i.e., 480 packets per minute), the packet loss ratio of the CoAR, ECRM, and RPL are about 20%, 27.6% and 50%, respectively. The reason behind the better performance of CoAR is, it because it reacts more precisely to congestion than other protocols.

**Figure 8:**
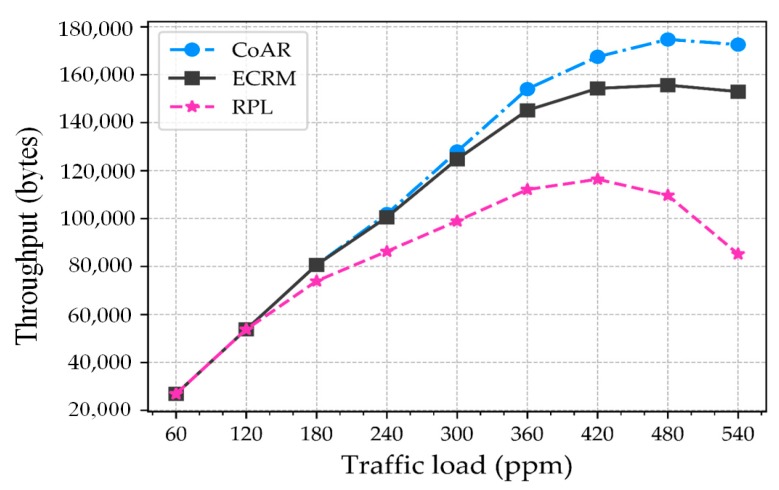
Throughput with respect to the traffic load.

**Throughput.** It is defined as the total amount of data bytes received at the root node in unit time. Figure 8 shows the throughput of the protocols. The result shows that even though the throughput of all protocols increases with the traffic load, the variation is noticed under high traffic loads. CoAR, and ECRM achieve higher throughput with increasing traffic load compared to RPL. This reveals that both resource control algorithms can work effectively in the network with a higher load. On the other hand, RPL lowers the throughput after traffic load exceeds 360 packets per minute. The reason is that RPL does not utilize the network resource control strategy to alleviate the congestion. CoAR achieves the highest throughput at a high traffic load of 480 packets per minute. In contrast, ECRM achieves the lower throughput for the same data generation rate. The figure also depicts that when the traffic load is 480 packets per minute, the throughput of the network achieved by CoAR is 8.87% greater than ECRM.

**Figure 9:**
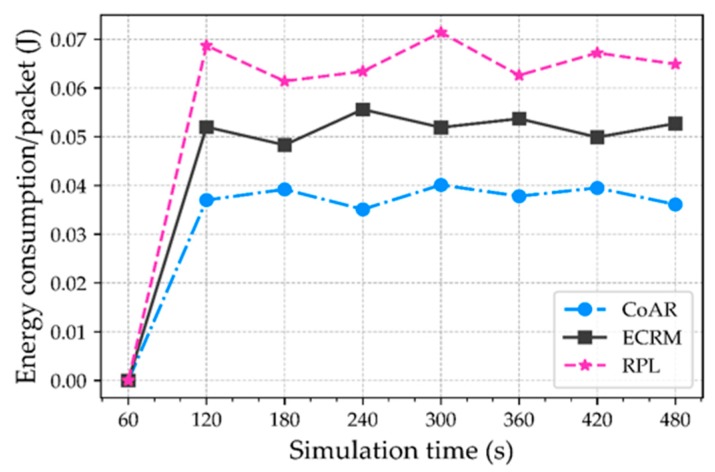
Network energy consumption.

**Energy consumption.** The energy consumption due to the transmission and reception of per successfully delivered packet from source to the destination is considered. The energy consumption of nodes in the network is shown in Figure 9. We note that the energy consumption of CoAR in the network is significantly lower than the others. Because CoAR loses fewer packets at the buffer and minimizes the number of retransmissions. On the other hand, ECRM and RPL dissipate energy by transmitting and receiving packets which are lost due to the buffer overflow.

### 5.3. Impacts of Varying Number of Nodes

In this section, we measure the different dimensions of LLNs to test the scalability of the protocol. The performance of the protocols varies according to the network size. In this regard, we analyze the influence of variation in network size. For this, we vary the number of deployed nodes in the network from 16 to 41. The traffic load to each network is kept at 480 packets per minute. The rest of the parameters remain the same.

**Figure 10:**
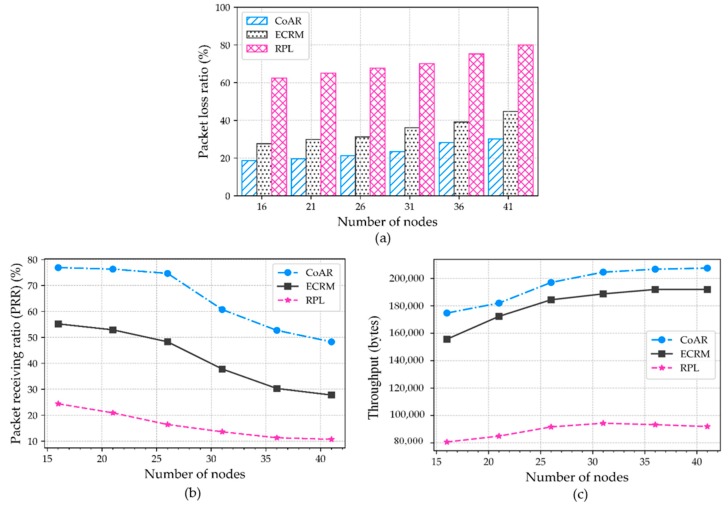
Data reliability over different number of nodes: (**a**) Packet loss ratio; (**b**) Packet receiving ratio; and (**c**) Throughput in bytes.

Figure 10a shows that CoAR maintains relatively low packet loss as compared to other protocols. With the growth of network size, more traffic flows can inject into the network and lead to congestion. Nevertheless, CoAR can change their congested parents to keep the queue utilization lower and bypass congested path, so that the lower packet loss rates are achieved. In contrast, with the increasing number of nodes, the packet loss ratio in RPL is higher than ECRM and CoAR. Because the default objective function in RPL cannot change the parents suffering from severe congestion. On the other hand, the packet loss ratio in ECRM marginally improved as compared to RPL; however, it is still not satisfactory for the proposed application scenario. As a result, at the network size of 16 and 41, CoAR outperforms RPL by 43.7% and 51.8% respectively.

As the number of nodes increases in the network, CoAR gets the opportunity to explore more alternate parents to forward a large number of packets to the destination and thus improves the throughput. Figure 10b shows the inclination in PRR with an increased number of nodes in the network. When there are 26 and 41 nodes in the network, the maximum PRR of the protocols is approximately 74.6% and 48.2% in CoAR, 48.3% and 27.7% in ECRM, and 16.4% and 10.7% in RPL, respectively. For a varying number of nodes, CoAR maintains the average of 23.2% and 48.7% better performance than ECRM and RPL, respectively.

As depicted in Figure 10c, for the increased number of nodes, CoAR improves overall throughput as compared to the protocols. When the network size is higher, the network throughput achieved by CoAR is 8.12% greater than ECRM. Unlike the protocols, CoAR dynamically set the *A_τ_* and allows the affected nodes to avoid persistent congestion situation. Besides this, *NI* metrics check the nodes vicinity during the parent selection, especially when network size is increased. Therefore, we notice that as the number of nodes increases, the CoAR performs better because of its resource control mechanism can operate properly. The RPL also marginally improved, but its performance is very low in terms of satisfying the application scenarios.

### 5.4. Impacts of Bursty Traffic Arrivals

**Figure 11:**
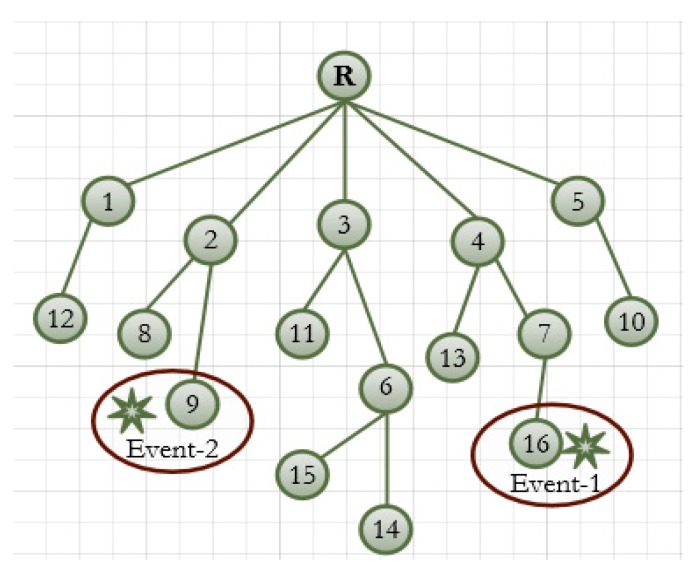
Sudden event occuring network topology.

In this part, we illustrate the importance of responsiveness under a highly dynamic traffic model. Here, we consider the impact of dynamic traffic bursts at different time instants in an industrial monitoring network. Figure 11 shows a network topology and two source nodes suffering from an unusual event occurring in the monitoring region. The occurrence of the two events and their burst periods is given in Table 3. We assume that the event occurs near sensing node 9 and 16 during the time interval as described. Only sensing node 9 and 16 can detect the occurrence of events. Moreover, the event reporting frequency of the sensing node is greater than the continuous monitoring reporting frequency. Furthermore, every event injects data traffic with a rate of 16 packets every second, and there are two bursts for each event.

**Figure 12:**
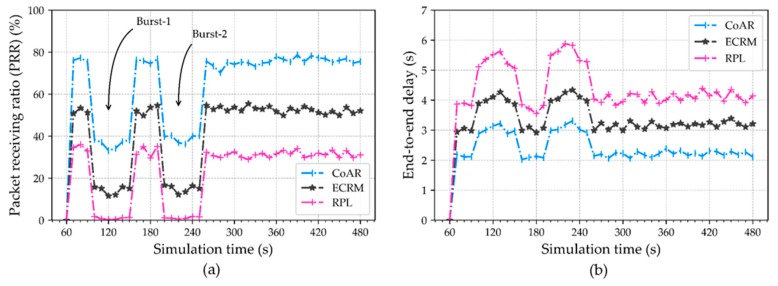
Impacts of bursty traffic arrival on reliability at the root node in the different time interval: (**a**) Packet receiving ratio; and (**b**) Average end-to-end delay during the burst period.

In Figure 12a, we observe that the adaptation capability of CoAR is higher than that of other approaches. Along with ensuring steady flow under low traffic loads, the CoAR is able to retain higher reliability (the packet receiving rate at root node) than ECRM and RPL even at bursty traffic durations. CoAR can effectively tolerate the burst because of its efficient congestion detection scheme and queue monitoring policy. As shown in the figure, we can note that the improved reliability of the CoAR is highlighted in terms of receiving packet rate at the time of two bursts (100~150 s, 200~250 s interval). This superior performance can be attributed to mainly, CoA-OF and adaptive congestion detection scheme. When the event occurs, the sensing node injects heavy traffic in the network and the queue occupancy of the parent increase sharply. As RPL does not have an efficient scheme to handle a large number of burst packets well, the packets are backlogged at the parent node in a very short interval. Therefore, RPL enters in an overloaded condition, which results in queue loss and delay hence the performance degrades. The ECRM is prone to congestion situation due to static congestion detection scheme and consecutive buffer overflow-based parent change policy, resulting in degraded network performance. Thus, dynamic traffic arrival, which is often a common phenomenon in industrial monitoring IoT application, can be better addressed and accommodated by our proposed scheme.

Figure 12b shows the average end-to-end delay. When the burst traffic arrives in the buffer, the overflow of packets is more likely to happen, leading to retransmission and queue lost. As shown in the figure, during the burst period, the amount of delay using CoAR is the least compared to other protocols. CoAR uses the congestion degree to determining the congestion situation adaptively which indicates the trend of queue fluctuations and thus smooths the burst traffic arrivals as much as possible. As a result, it achieves the better reliability and decreases packet loss. This, in turn, leads to a decrease in end-to-end delay.

## 6. Discussion

Before drawing conclusions, we first highlight the key points in our proposition. Then, we further discuss some design limitations and extensions of CoAR for future investigation.

Based on the simulation results and performance analysis, it is clear that CoAR has overall superior performance in comparison with other protocols. The performance supremacy, in fact, comes from the congestion control strategy along with other factors, which can be summarized as follows:

- The advantage of routing metrics used in CoAR is to utilize the network resources effectively. *QU* metric is considered to prefer the nodes that have more free space in their buffer, *ETX* to provide reliability and *RE* to select the node having high energy. The tie-breaking metric *NI* is considered as a criterion to select a preferred parent on a greedy basis.
- CoAR adopts the congestion-aware objective function as the parent selection framework for the decision-making problem within the congestion. To rank the alternative parents of a node, it combines the multiple routing metrics and the decision problem is solved by using the TOPSIS method. In addition, trickle algorithm in CoAR is aware of congestion and thus reset the timer as soon as congestion is detected. Actually, to achieve a balance between control overhead and fast recovery, CoAR send the congestion notification using DIO message.
- The adaptive threshold significantly utilizes the buffer capacity of a node hence detects the congestion timely. Additionally, the other factors that enhance the efficacy of CoAR is an appropriate adaptation of the weighting of routing metrics which accommodates to the performance criteria of the intended IoT application of emergency scenario.

While CoAR proved its efficiency in the simulation tests, there are still some aspects that need further investigation. In our congestion detection mechanism, the smoothing factor *α* and *β* were constant for the outgoing and incoming traffic respectively. The smoothing factors are used to adjust the level of smoothing to traffic measurement. The selection of this factors could be optimized and made it dynamic using some latest techniques such as fuzzy logic. However, it should be reasonable for resource constrained devices. Even though IoT application of emergency scenario might consist of mobile nodes, CoAR is described considering only a static network topology. However, we leave the optimization of CoAR in a mobile environment for future investigations.

Providing security in RPL based network is challenging because of the resource constrained devices and LLN characteristics. Many of the vulnerabilities of LLNs will create the transition to IoT, mainly with RPL attacks. The security issues in RPL has been analyzed by the research community mainly in security attacks, such as DODAG version attacks [37], Suppression attack [38], Routing attacks [39], and Hatchetman attack [40]. Even though security is an important issue in routing protocol, it is out of the scope in this work. However, in future work, we plan to investigate the DIO suppression attack because it can severely degrade the routing service in CoAR. This attack induces victim nodes to suppress the transmission of DIO, which are necessary to build the route to alleviate congestion.

## 7. Conclusions

In this paper, we propose a congestion-aware routing mechanism whereby the congestion is alleviated by using the resource control method that suitably detects the congestion based on the present and past traffic trends. To solve the parent selection problem within congestion, we have defined the framework as the multi-criteria decision-making problem, which is solved by using the TOPSIS method. Different routing metrics are used as the parent selection criteria so that the CoAR can balance the workload of nodes in industrial monitoring network to achieve the desired QoS. The congestion detection mechanism ensures the affected nodes to avoid transient congestion situation, meantime it renders the best parent selection to create an alternate path towards the root in order to control persistent congestion situations so that maximize the packet reception rate. Additionally, along with ensuring steady flow under low traffic loads, the CoAR is able to maintain higher reliability even at bursty traffic durations. Overall, to satisfy the IoT application requirements, CoAR is aware of network situation under heavy traffic as well as the unique characteristic of LLNs and IEEE 802.15.4. Extensive simulation tests are conducted to verify the performance. The simulation results validate that CoAR improves the PRR, end-to-end delay, packet loss ratio, throughput, and energy consumption, as compared to the existing protocols.

## Figures and Tables

**Table 1:** Comparative characteristic of protocols.

Protocol	Congestion Alleviation	Congestion Detection	Congestion Notification	Traffic Type
Bird flock [17]	Resource control	Buffer occupancy	No	Continuous
GTCC [18]	Traffic and resource control	Difference between packet generation rate and packet service rate	Implicit	Continuous
DCCC6 [19]	Traffic control	Buffer occupancy	Implicit/Explicit	Continuous
QU-RPL [5]	Resource control	Buffer overflow	Implicit	Continuous
GTCCF [20]	Traffic control	Ratio between total receiving rate and total forwarding rate	Implicit	Continuous
CA-RPL [21]	Resource control	No	Explicit	Continuous
ECRM [25]	Resource control	Buffer overflow	Implicit	Continuous
CoAR	Resource control	Buffer occupancy, ratio of incoming traffic rate over outgoing traffic rate	Implicit	Continuous, Event- based

**Table 2:** Simulation parameters setting.

Parameters	Value
Topology area	200 × 200 m^2^
Transmission mode	Storing
Transmission range	60 m
Interference range	70 m
Adaptation	6LoWPAN
Radio model	Unit Disk Graph Medium (UDGM)
Loss model	Distance loss
Mote type	Tmote sky
Queue size	12 packets
Queue type	FIFO
*I_min_*/*I_doubling_*	0.75 s/8 s
Data link	CSMA; ContikiMAC (RDC)
Channel check rate	16 Hz

**Table 3:** Traffic bursts activated during different time intervals of the simulation period.

	Event 1	Event 2
Burst 1	100 s~130 s	120 s~150 s
Burst 2	200 s~230 s	220 s~250 s
